# Honeybee wings hold antibiofouling and antimicrobial clues for improved applications in health care and industries

**DOI:** 10.3934/microbiol.2023018

**Published:** 2023-04-03

**Authors:** Akamu J. Ewunkem, A'lyiha F. Beard, Brittany L. Justice, Sabrina L. Peoples, Jeffery A. Meixner, Watson Kemper, Uchenna B. Iloghalu

**Affiliations:** 1 Department of Biological Sciences, Winston Salem State University, Winston-Salem, North Carolina, USA; 2 Department of Clinical Sciences, Winston Salem State University, Winston-Salem State, North Carolina, USA; 3 Department of Biology, North Carolina A and T State University, Winston-Salem State, North Carolina, USA; 4 Department of Biology, Guilford College, Greensboro, North Carolina, USA

**Keywords:** honeybee, bacteria, antimicrobial, anti-biofouling, wings, workers

## Abstract

Natural surfaces with remarkable properties and functionality have become the focus of intense research. Heretofore, the natural antimicrobial properties of insect wings have inspired research into their applications. The wings of cicadas, butterflies, dragonflies, and damselflies have evolved phenomenal anti-biofouling and antimicrobial properties. These wings are covered by periodic topography ranging from highly ordered hexagonal arrays of nanopillars to intricate “Christmas-tree” like structures with the ability to kill microbes by physically rupturing the cell membrane. In contrast, the topography of honeybee wings has received less attention. The role topography plays in antibiofouling, and antimicrobial activity of honeybee wings has never been investigated. Here, through antimicrobial and electron microscopy studies, we showed that pristine honeybee wings displayed no microbes on the wing surface. Also, the wings displayed antimicrobial properties that disrupt microbial cells and inhibit their growth. The antimicrobial activities of the wings were extremely effective at inhibiting the growth of Gram-negative bacterial cells when compared to Gram-positive bacterial cells. The fore wing was effective at inhibiting the growth of Gram-negative bacteria compared to Gram-positive samples. Electron microscopy revealed that the wings were studded with an array of rough, sharp, and pointed pillars that were distributed on both the dorsal and ventral sides, which enhanced anti-biofouling and antimicrobial effects. Our findings demonstrate the potential benefits of incorporating honeybee wings nanopatterns into the design of antibacterial nanomaterials which can be translated into countless applications in healthcare and industry.

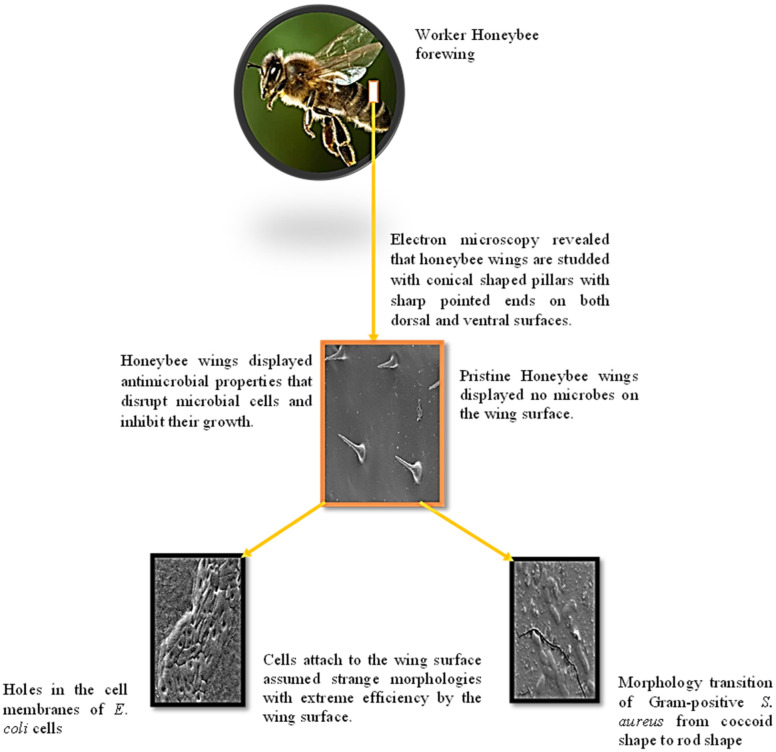

## 1. Introduction

In recent years the number of infections associated with antimicrobial resistance has increased and is emerging as one of the leading public health threats of the 21st century [1],[2]. The accelerated spread of antibiotic resistance is due to the inappropriate and excessive use of antibiotics in the past few decades [3]. Nanomaterials are a promising means in curbing the use of antibiotics due to their mechanism of preventing bacterial attachment to kill bacteria [4]. The bactericidal mechanisms associated with nanomaterials are generally divided into two major categories. Nanomaterials may act as carriers, delivering antibiotics to bacteria increasing drug potency and minimizing overall drug exposure. Also, nanomaterials can generate lethal damage to microbes through a physical process that destroys extracellular polymeric substances (EPS) using either enzymes or mechanical forces [4]. Insect wings have nanometer-sized structures with the potential to break EPS [5].

Insect wings are membranous, parchment-like, heavily sclerotized, and can be fringed with long hairs or covered with scales [6]. The wings enable a myriad of ecologically important behaviors including flight, thermal collection, gyroscopic stabilization, sound production, fellow species recognition, sexual overtures and contact, and protective cover [7]–[9]. Various insect wings such as cicada (e.g., *Psaltoda claripennis*) (Ashton, 1921), damselfly fly (e.g., *Calopteryx haemorrhoidalis* (Vander Linden, 1825) and dragonfly (e.g., *Pantala flavescens* (Fabricius, 1798)) maintain contaminant-free status and possess antimicrobial properties [10]–[12]. It has been established that the antimicrobial properties of the wings of cicada, damselfly fly, and dragon flies are mediated by the physical nanoprotrusions or nanopillars found on the wing surface [13],[14]. The nanopillars are known to damage bacteria by physically puncturing the bacterial envelope, inducing deformation [15]–[18]. These nanoarchitectures are effective against a wide range of bacteria including *Pseudomonas aeruginosa, Escherichia coli, Branhamella catarrhalis*, and *Pseudomonas fluorescens, Bacillus subtilis, Pseudococcus maritimus*, and *Staphylococcus aureus* [13],[15],[19],[20]. Although the antibiofouling (self-cleaning ability) and antimicrobial efficiency of cicada, damselfly fly and dragonfly has been widely reported, the effectiveness of the wings of honeybees against bacteria, however, is yet to be established.

Honeybees *Apis mellifera* (Linnaeus, 1758) are considered as super-generalist pollinators in agricultural systems [21],[22]. These social and hardworking insects can produce royal jelly, beeswax, propolis, venom and honey for various nutritional and medicinal purposes [23],[24]. To date, few studies have concentrated on the antimicrobial activity of honeybee products, which have been used extensively since ancient times, as documented in Egyptian, Roman, Chinese and Persian literatures [25]. The antimicrobial activity of honey is mediated by the activation of the enzyme glucose oxidase which oxidizes glucose to gluconic acid and hydrogen peroxide (H_2_O_2_), a potent antimicrobial agent [26]–[28].

In contrast, the topography of honeybee wings has received less attention. The role topography plays in antibiofouling, and antimicrobial activity of honeybee wings has never been investigated. In this study we investigated the antibiofouling and antimicrobial activities of the wings of non-reproductive female honeybees, also called “workers.” The workers were selected for this study because they are by far the most numerous castes in the hives and perform all the work needed to keep the colony fed and healthy. As the workers carry out these tasks inside and outside the hive, they could theoretically be exposed to a wide variety of pathogens providing an opportunity for pathogens to spread from the workers to the other castes, e.g., drones and queens [29]. The results of this study will help to explain why honeybee workers are such successful foragers. Also, these findings could lead to the development of self-cleaning and antimicrobial surfaces with myriad applications including hospital surfaces, filters for high-risk indwelling devices (e.g., foley catheters), agricultural and food service applications etc.

## 2. Materials and methods

### 2.1. Honeybee collection and sample preparation

**Figure 1.**
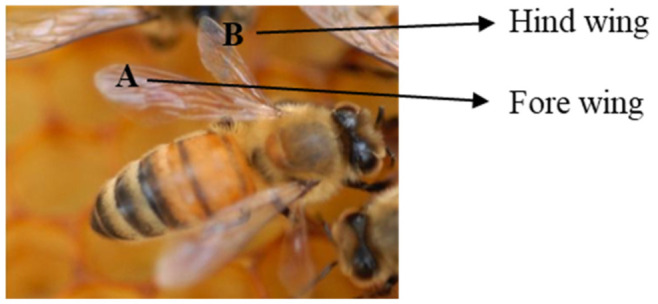
Forewing and hindwing of a worker honeybee (*Apis mellifera*). Images of the forewing (A) and hindwing (B).

Thirty mature honeybee (*Apis mellifera*) workers were collected from Dr. Jeffery Andrew Meixner's beehive, in Winston Salem, North Carolina USA. The wings of honeybee are arranged in two pairs, connected by a row of hooks on the back wing [30] (Figure 1). The fore wings (~0.8 cm) are much larger than the hind wings (~0.4 cm). Both fore and hind wings of each specimen were incised by a scalpel from the body of the insect meticulously (avoiding damage to their surfaces) and stored at room temperature in sterile polystyrene Petri dishes (Fisher Scientific) until required.

### 2.2. Scanning electron microscopy

Scanning electron microscopy (SEM) was used to observe the dorsal and ventral surfaces of pristine honeybee wings for the presence of microbes. SEM has been used intensively to revealed micro- and nano scales on wing surfaces [13]–[15],[30]. The wings were glued onto copper pin mounts with a double-sided carbon adhesive tape, and SEM imaging was done using a scanning electron microscope (Carl Zeiss Auriga-BU FIB FESEM) from the Joint School of Nanoscience and Nanoengineering, Greensboro, North Carolina, USA.

### 2.3. Bacterial strains, growth, and sample preparation

*Escherichia coli 1946* (ATCC 25922), *Staphylococcus aureus* (ATCC 25923), *klebsiella pneumoniae* NCTC 9633 (ATCC 13883) and *Micrococcus luteus* (ATCC 4698) were obtained from the collection of the Department of Biological Sciences, Winston Salem State University. The isolates were checked for purity and maintained in slant of Nutrient agar (ThermoFisher Scientific, Waltham, MA, USA). Nutrient broth (NB) was used as the growth medium for the bacteria. Prior to the assays, the bacteria strains were cultured at 37 °C in 5 mL of NB media with shaking (120 rpm).

### 2.4. Antimicrobial activity: 24-hour growth

Antibacterial activities of both hind and forewings of honeybees were tested using *Escherichia coli, Staphylococcus aureus, Klebsiella pneumoniae and Micrococcus luteus*. These strains were cultured overnight in NB media (ThermoFisher Scientific, Waltham, MA, USA) in clear 96-well plates at a final volume of 200 µL for 24 h at 37 °C in a shaker (120 rpm). To start the twelve-row by eight-column plate was divided into 2 sections. One column was designated the “experimental” column and one was designated the “control”. With the help of a sterilized pair of forceps, each wing (intact wing) was meticulously transferred to a well. Then, 100 µL of NB media was placed into each cell of the experimental and control column down the 8 rows: negative control (media only (M)), positive control (media + cells (CM)), experimental well 1 (fore wing (FW) + media + cells), experimental well 2 (hind wings (H) + media + cells), experimental well 3 (hind wings + fore wings (FHW) + media + cells) and experimental well 3 (fore wing (FW) + media ). The assays were carried out in triplicate and bacterial growth was assessed by measuring turbidity at 600 nm for hours 0 and 24 h, using a 98-well plate format Glomaxmulti plate reader (Promega, USA). To determine the bacterial load (CFU/mL), cells from each treatment were collected after 24 hours of incubation. Samples were diluted 4 times with sterile NB and 10 µL of each dilution was spread on NA agar plates with the help of sterile L-Shaped cell spreaders. The plates were incubated at 37 °C for 24 hours. Colonies on each plate were then counted. The CFU/mL was calculated as CFU/mL = n° colonies x dilution factor). After 24 hours of incubation the forewings were aseptically removed from the 96-well plates for SEM as described above.

### 2.5. Statistical analysis

Statistical Analysis Statistical analyses were performed using GraphPad Prism version 7.00 (GraphPad software, La Jolla, USA). The antimicrobial activities were analyzed using Student's T-tests. Statistical differences between groups were considered significant at a p-value < 0.05.

## 3. Results

### 3.1. Scanning electron microscopy of pristine wings

**Figure 2.**
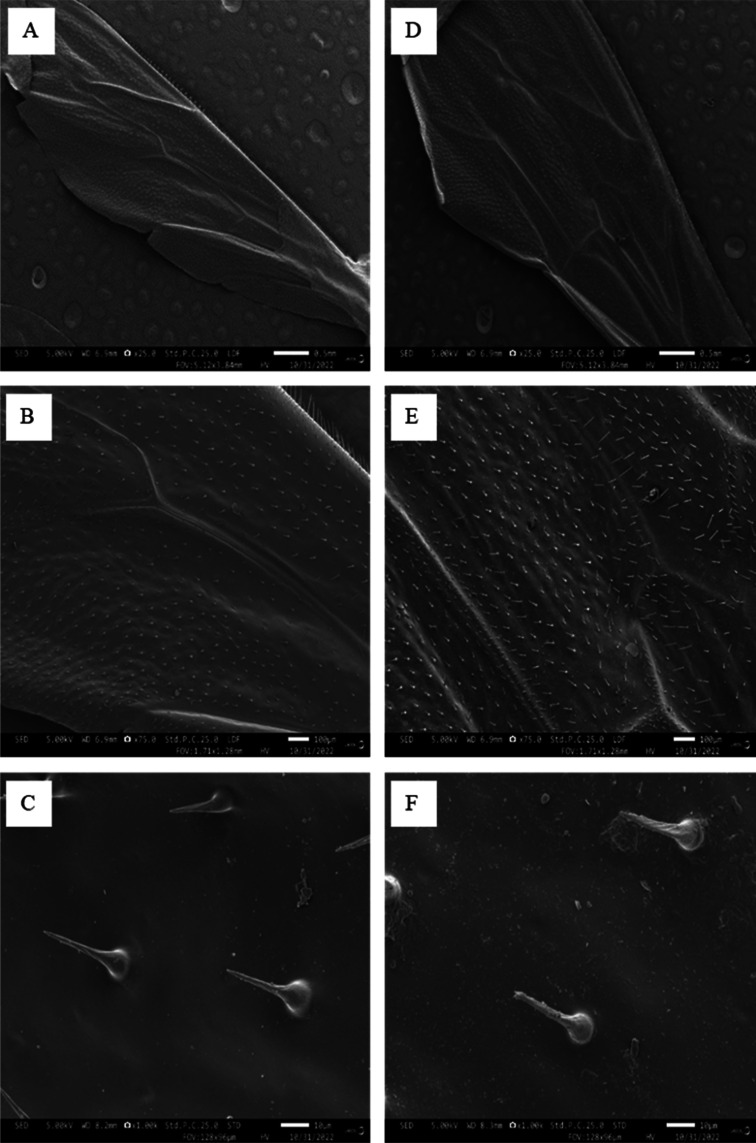
Scanning electron microscopy (SEM) of dorsal surface of a worker honey bee (*Apis mellifera*) fore wing (A; B; C) and hind wing (D; E; F) surface topography at different magnifications: (A and D) at x 25; (B and E) at x 75 and (C and F) at x10000.

The surface structures of the hind and fore wings of adult honeybee workers from the wild were extensively characterized by scanning electron microscope imaging technique. Forewing and hindwing surfaces were covered by an array of conical shaped pillars with sharp pointed ends on both dorsal and ventral surfaces (Figure 2). The array consisted of closely packed pillars with a base diameter 7.0 ± 0.25 µm, a height of 20.0 ± 2.22 µm, and spacing of 65.0 ± 3.41 µm apart from each other. Furthermore, the pillars were arranged in a manner similar to that of a parallelogram. Taken together these results show that honeybee workers' wings possess microscopic spikes that may defend against microbes.

### 3.2. Antimicrobial activity: 24-hour growth

**Figure 3.**
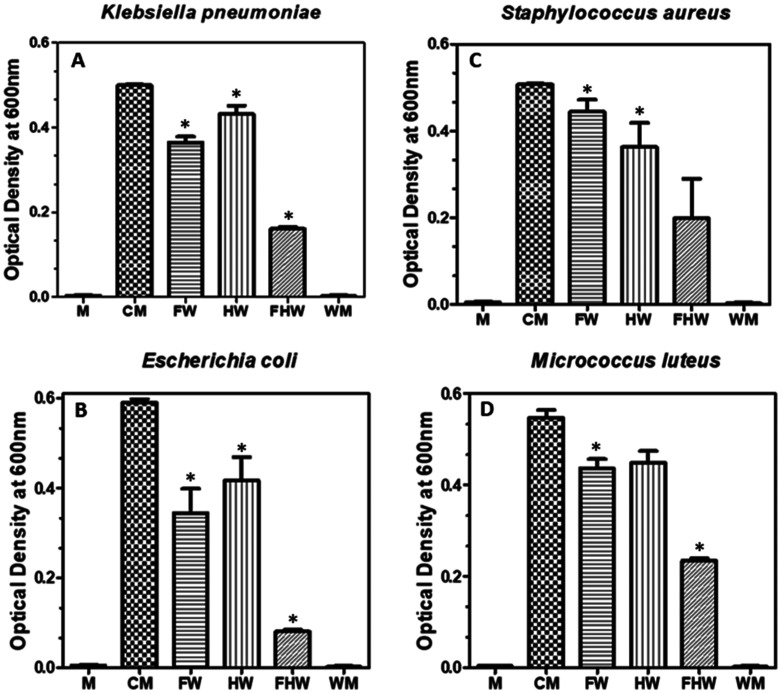
Antimicrobial efficacy of a worker honeybee (*Apis mellifera*) wings against bacteria. (A) *Klebsiella pneumoniae*; (B) *Escherichia coli*; (C) *Staphylococcus aureus*, and (D) *Micrococcus luteus*.

**Figure 4.**
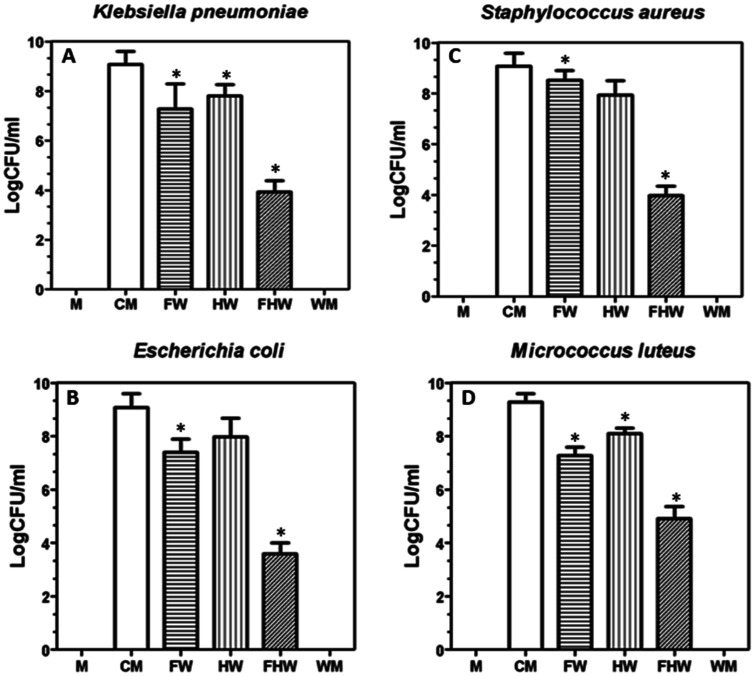
Viable cell number reduction of bacteria, expressed as log10 cfu/mL, after overnight exposure to honeybee worker wings.

According to Figure 3 microbial growth did not occur in the one of experimental wells (wings + media) suggesting self-cleaning or antibiofouling. Meanwhile, on the negative control (Cells + Media only) microbial growth was recorded. The results of the antimicrobial tests on the worker honeybee wings against bacteria showed that stronger antimicrobial activity was against the Gram-negative bacteria (*E. coli* and *K. pneumoniae*) than against the Gram-positive bacteria (*S. aureus* and *M*. *luteus*) (Figure 4). The fore wing (FW) was extremely effective at slowing the growth of Gram-negative bacteria when compared to Gram-positive samples. The presence of a single forewing significantly reduced the growth of *E. coli* and *K. pnuemoniae* when compared to the control samples (i.e., cells and media) (CM) (Figure 4A, 4B). In comparison, the hind wings showed no significant reduction in optical densities of *E. coli and K. pnuemoniae* (Figure 4A, 4B). This pattern was not observed for the Gram-positive bacterial cells. The hind wing significantly decreased the growth of *S. aureus* when compared to the fore wing (Figure 4C). The effects of the forewing and hind wing on the growth of *M. luteus* were comparable (Figure 4D). Finally, we assessed the bacterial growth in the presence of both hind and fore wings (FHW). We observed a statistically significant reduction in the growth of all the bacteria with a greater reduction in Gram-positive (*E. coli* and *K. pneumoniae*) (Figure 3).

For *S. aureus*, *K. pneumoniae*, *E. coli*; *S*. aureus, and *M. luteus*, significant reductions in CFU relative to controls were observed on the wings following 24-h incubation (Figure 4), corroborating the antimicrobial efficacy of a worker honeybee (Figure 3).

**Figure 5.**
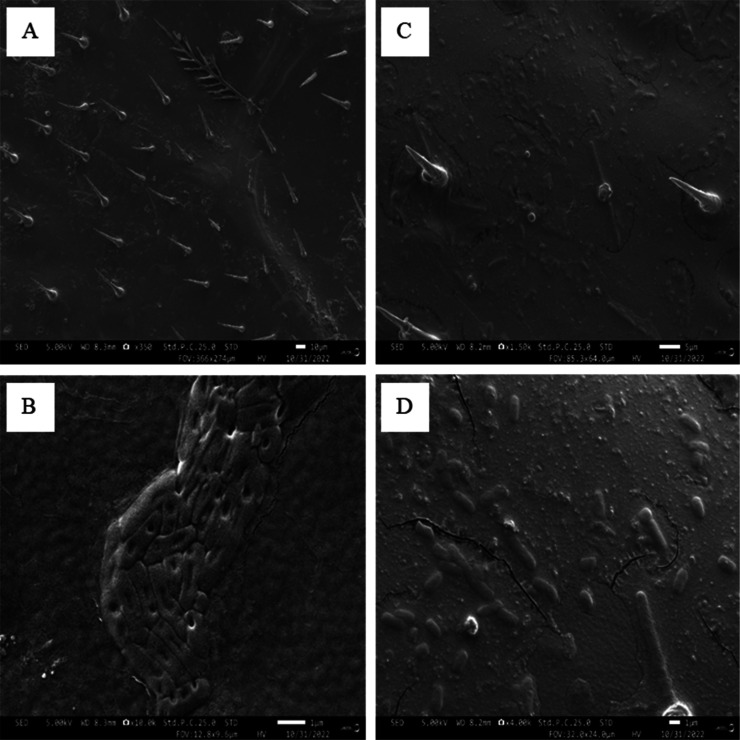
SEM images of forewings exposed to (A) *E. coli* and (B) *S*. *aureus*.

The bacterial cells were found to be capable of adhering effectively onto the surface of the honeybee fore wings; however, those cells that were able to attach to the surface assumed strange morphologies with extreme efficiency by the wing surface. The wing was able to punch holes in the cell membranes of Gram-negative *E. coli* cells (Figure 5A, B) while there is a morphology transition of gram-positive *S. aureus* from coccoid shape to rod shape (Figure 5C, D). This may be a survival strategy that evolved to help these bacteria survive in less-than-ideal conditions. Altogether, our results reveal that honeybee worker wings exhibit antibacterial properties against Gram-negative bacteria, *E. Coli, K. pneumoniae* and Gram-positive, *S. aureus* and *M. luteus*. Additionally, these wings are self-cleaning.

## 4. Discussion

Some insects such as cicada, damselfly, and dragonfly are known to have antimicrobial and antibiofouling potential mediated by mediated by the physical nanoprotrusions (known as nanopillars) found on the wing surface [15],[31],[32]. To the best of our knowledge, there have been no studies that demonstrate the presence of array structures on the wings of honeybees that confer antibiofouling and antimicrobial activities. This is consequential because honeybees are arguably the most important managed species for agricultural pollination across the world.

In this study, we demonstrated that honeybee workers possess surface features that significantly increased antibiofouling and antimicrobial activities against the Gram-negative bacteria (*E. coli* and *K. pneumoniae*) and Gram-positive bacteria (*S. aureus* and *M. luteus*). Analysis of these wings using SEM showed highly ordered closely packed 20.0 µm tall spike structures, called “nanopillars”, that are spaced approximately 170 nm apart. The exact mechanisms on how nanopillars inhibit the growth of bacteria is yet to be understood and resolutions/answers have long tantalized researchers. However, recent studies involving titanium oxide suggest that the array and evenly distributed nanopillars on the wings of cicada and dragon flies exhibit antimicrobial activities by penetrating bacterial cells, causing the cellular membranes to stretch, and cells to rupture [33].

The microstructure or “nanopillars” of pristine honeybee worker wings contribute to rugged surface increasing the roughness of wings and the likelihood of anti-biofouling defined as self-cleaning ability to prevent contamination by particles such as dust, spores, and bacteria. Our investigations of the worker honeybee wings from the wild revealed that the wing surface was effective at killing or inhibiting the growth of adhered bacteria (Figure 3). Hydrophobic surfaces are famously known to inhibit the attachment of bacterial cells and there could be a relationship between hydrophobicity, surface roughness and antibiofouling that work in a concerted fashion to produce antimicrobial effects. Studies have shown that hydrophobic property associated with honeybee wings is attributed to the coupling of rough surface microstructure and hydrophobic protein constituents [34],[35]. The main constituent of the hydrophobic polypeptide belongs to the 60 S acidic ribosomal protein P1 and is reported to inhibit infectious bacteria due to an increase in intracellular reactive oxidative species (ROS), which, in turn, could affect membrane integrity and cause cell death [36],[37].

In this study, Gram-negative bacteria (*K. pneumoniae*, *E. coli*) were more susceptible to the bactericidal effect of the wings compared to Gram-positive bacteria (*S. aureus*, and *M. luteus*). These observations are consistent with reports made in previous literature that employed either naturally occurring bactericidal topographies or replicas derived from the natural counterparts. Gram-Positive bacteria are armed with rigid membranes, making them more resistant to the lethal effects of the wings while Gram-negative bacteria with more elastic membranes are more susceptible [38]. This provides further evidence in support of the assertion made in other studies using cicada wings that the bactericidal activity is mechanical in nature [13],[15] where cicada wings were more effective in killing Gram-negative bacteria (i.e., *Branhamella catarrhalis, Escherichia coli*, and *Pseudomonas fluorescens*) than Gram-positive bacteria (*Bacillus subtilis*, *Pseudococcus maritimus*, and *Staphylococcus aureus*) [13]. This suggests that the primary factor determining the susceptibility of cells to the wing surface was the rigidity of the cells.

Honeybee wing surfaces caused morphologic and structural variation in Gram-negative and Gram-positive bacteria. Damage to *E. coli* cells was characterized by holes in the membranes while *S. aureus* cells increased in length and transformed from a coccoid shape to rod shape. The holes in the *E. coli* cells were absent in *S. aureus* cells because of the presence a thick peptidoglycan which is known to confer strength and rigidity. An increase in length of *S. aureus* could be due to stress, which results in the production of ROS as observed in *Lactobacillus spp*, in *M. tuberculosis,* and *M. smegmatis* [39]–[41].

The nanoarchitecture and self-cleaning ability of the worker honeybee's wings serves lifestyle requirements, similar to migratory habits and foraging behavior. Honeybees have an organized system of dividing labor amongst each other in their societies [42]. In this paper, we will focus on the function of the workers' wings. The tasks delegated among the workers within a colony are based on the age of the individual and on the needs of the colony [43]. Immediately after emergence, juvenile workers clean cells and feed older larvae while mature worker bees perform indoor duties inside the hive [44],[45]. Thereafter, they become foragers, collecting water, pollen, nectar and propolis. The versatile activities of workers expose them to pathogens with significant effects such as paralysis, respiratory failure, and mortality of target (workers) and non-target (e.g., drones and queens) individuals. The *A. mellifera* workers need a highly adapted system as the tasks they perform during their lifespan expose them to distinct pressures from pathogenic exposure [46]. In general, the first line of defense against most pathogens is the cuticle, a preventive barrier designed to prevent or retard the entry of pathogens into the haemoceol [47],[48]. Like all insect body parts, the wings are made from cuticle, which is the second most common natural material in the world. In honeybees, the production of cuticle protein CPR14 plays important roles for cuticle maturation and maintenance, thus providing an effective protection against pathogens in adult workers [43],[49]. Therefore, the combination of the physical and chemical properties of the worker wings are efficient in creating a hostile niche for bacterial colonization and growth.

## 5. Conclusion

We have demonstrated evidence that worker honeybee wings exhibit a wide range of anti-biofouling and antimicrobial properties. The tested Gram-negative bacteria (*Klebsiella pneumoniae, Escherichia coli*) were more susceptible to the bactericidal effect of the wings compared to Gram-positive bacteria (*Staphylococcus aureus, and Micrococcus luteus*). These properties may be attributed primarily to a physical mechanism, but we have not ruled out any potential biochemical mechanisms. Topographical analysis of the wing surfaces showed regularly spaced conical shaped pillars with sharp points. The features of honeybee worker wings can inspire a new approach in the development of novel functional surfaces that possess an increased resistance to bacterial contamination and infection. These findings have a multitude of potential applications in industry, medical technology, clothing manufacturing, etc. Further study is needed to actualize these applications.

## References

[1] Lesho EP, Laguio-Vila M (2019). The slow-motion catastrophe of antimicrobial resistance and practical interventions for all prescribers. Mayo Clin Proc.

[2] Murray CJL, Ikuta KS, Sharara F (2022). Global burden of bacterial antimicrobial resistance in 2019: a systematic analysis. Lance.

[3] Saha M, Sarkar A (2021). Review on multiple facets of drug resistance: a rising challenge in the 21st century. J Xenobiot.

[4] Gao W, Zhang L (2021). Nanomaterials arising amid antibiotic resistance. Nat Rev Microbiol.

[5] Oopath SV, Baji A, Abtahi M (2023). Nature-inspired biomimetic surfaces for controlling bacterial attachment and biofilm development. Adv Mater Interfaces.

[6] Wagner GP (2007). The developmental genetics of homology. Nat Rev Genet.

[7] Linz DM, Tomoyasu Y (2018). Dual evolutionary origin of insect wings supported by an investigation of the abdominal wing serial homologs in Tribolium. Proc Natl Acad Sci.

[8] Deora T, Sane SS, Sane SP (2021). Wings and halteres act as coupled dual oscillators in flies. Elife.

[9] Salcedo MK, Socha JJ (2020). Circulation in insect wings. Integr Comp Biol.

[10] Truong VK, Geeganagamage NM, Baulin VA (2017). The susceptibility of *Staphylococcus aureus* CIP 65.8 and *Pseudomonas aeruginosa* ATCC 9721 cells to the bactericidal action of nanostructured *Calopteryx haemorrhoidalis* damselfly wing surfaces. Appl Microbiol Biotechnol.

[11] Linklater DP, Juodkazis S, Rubanov S (2017). Comment on “bactericidal effects of natural nanotopography of dragonfly wing on *Escherichia coli*”. ACS Appl Mater Interfaces.

[12] Cheeseman S, Owen S, Truong VK (2018). Pillars of life: is there a relationship between lifestyle factors and the surface characteristics of dragonfly wings?. ACS Omega.

[13] Hasan J, Webb HK, Truong VK (2013). Selective bactericidal activity of nanopatterned superhydrophobic cicada *Psaltoda claripennis* wing surfaces. Appl Microbiol Biotechnol.

[14] Nowlin K, Boseman A, Covell A (2015). Adhesion-dependent rupturing of *Saccharomyces cerevisiae* on biological antimicrobial nanostructured surfaces. J R Soc Interface.

[15] Ivanova EP, Hasan J, Webb HK (2012). Natural bactericidal surfaces: mechanical rupture of Pseudomonas aeruginosa cells by cicada wings. Small.

[16] Pogodin S, Hasan J, Baulin VA (2013). Biophysical model of bacterial cell interactions with nanopatterned cicada wing surfaces. Biophys J.

[17] Jenkins J, Mantell J, Neal C (2020). Antibacterial effects of nanopillar surfaces are mediated by cell impedance, penetration and induction of oxidative stress. Nat Commun.

[18] Ivanova EP, Linklater DP, Werner M (2020). The multi-faceted mechano-bactericidal mechanism of nanostructured surfaces. Proc Natl Acad Sci.

[19] Heckmann TS, Schiffman JD (2019). Spatially organized nanopillar arrays dissimilarly affect the antifouling and antibacterial activities of *Escherichia coli* and *Staphylococcus aureus*. ACS Appl Nano Mater.

[20] Bandara CD, Ballerin G, Leppanen M (2020). Resolving bio–nano interactions of *E. coli* bacteria–dragonfly wing interface with helium ion and 3D-structured illumination microscopy to understand bacterial death on nanotopography. ACS Biomater Sci Eng.

[21] Dupont YL, Hansen DM, Olesen JM (2003). Structure of a plant-flower-visitor network in the high-altitude sub-alpine desert of Tenerife, Canary Islands. Ecography.

[22] Greenleaf SS, Kremen C (2006). Wild bees enhance honey bees' pollination of hybrid sunflower. Proc Natl Acad Sci.

[23] Fratini F, Cilia G, Turchi B (2016). Beeswax: A minireview of its antimicrobial activity and its application in medicine. Asian Pac J Trop Med.

[24] Rortais A, Barrucci F, Ercolano V (2021). A topic model approach to identify and track emerging risks from beeswax adulteration in the media. Food Control.

[25] Nader RA, Mackieh R, Wehbe R (2021). Beehive products as antibacterial agents: a review. Antibiotics.

[26] Bang LM, Buntting C, Molan P (2003). The effect of dilution on the rate of hydrogen peroxide production in honey and its implications for wound healing. J Altern Complementary Med.

[27] Bizerra FC, Da Silva PI, Hayashi MAF (2012). Exploring the antibacterial properties of honey and its potential. Front Microbiol.

[28] Kuś PM, Szweda P, Jerković I (2016). Activity of Polish unifloral honeys against pathogenic bacteria and its correlation with colour, phenolic content, antioxidant capacity and other parameters. Lett Appl Microbiol.

[29] Langlands Z, du Rand EE, Yusuf AA (2022). Functional response of the hypopharyngeal glands to a social parasitism challenge in Southern African honey bee subspecies. Parasitol Res.

[30] Ma Y, Ning JG, Ren HL (2015). The function of resilin in honeybee wings. J Exp Biol.

[31] Ivanova EP, Hasan J, Webb HK (2013). Bactericidal activity of black silicon. Nat Commun.

[32] Valiei A, Lin N, Bryche JF (2020). Mechano-bactericidal nanopillars require external forces to effectively kill bacteria. Nano Lett.

[33] Luo L, Zhou Y, Xu X (2020). Progress in construction of bio-inspired physico-antimicrobial surfaces. Nanotechnol Rev.

[34] Ivanova EP, Linklater DP, Aburto-Medina A (2021). Antifungal versus antibacterial defence of insect wings. J Colloid Interface Sci.

[35] Liang Y, Zhao J, Yan S (2017). Honeybees have hydrophobic wings that enable them to fly through fog and dew. J Bionic Eng.

[36] Arias SL, Devorkin J, Spear JC (2020). Bacterial envelope damage inflicted by bioinspired nanostructures grown in a hydrogel. ACS Appl Bio Mate.

[37] Hurtado-Rios JJ, Carrasco-Navarro U, Almanza-Pérez JC (2022). Ribosomes: the new role of ribosomal proteins as natural antimicrobials. Int J Mol Sci.

[38] Yao X, Walter J, Burke S (2002). Atomic force microscopy and theoretical considerations of surface properties and turgor pressures of bacteria. Colloids Surf B.

[39] Manina G, Dhar N, McKinney JD (2015). Stress and host immunity amplify *Mycobacterium tuberculosis* phenotypic heterogeneity and induce nongrowing metabolically active forms. Cell Host Microbe.

[40] Priestman M, Thomas P, Robertson BD (2017). Mycobacteria modify their cell size control under sub-optimal carbon sources. Front Cell Dev Biol.

[41] Ewunkem AJ, Rodgers LS, Campbell D (2021). Experimental evolution of magnetite nanoparticle resistance in *Escherichia coli*. Nanomaterials.

[42] Zhang X, Hao Y, Niu Q (2022). Division of labor among worker bees is associated with the lipidomic plasticity in their brains. Agriculture.

[43] da Luz GF, Santana WC, Santos CG (2022). Cuticle melanization and the expression of immune-related genes in the honeybee Apis mellifera (Hymenoptera: Apidae) adult workers. Comp Biochem Physiol Part B: Biochem Mol Biol.

[44] Moore AJ, Breed MD, Moor MJ (1987). The guard honey bee: ontogeny and behavioural variability of workers performing a specialized task. Anim Behav.

[45] Johnson BR (2005). Limited flexibility in the temporal caste system of the honey bee. Behav Ecol Sociobiol.

[46] Byhrø EMH, Salmela H, Vitlic A (2019). Different activation of immune-related genes in honey bee nurses and foragers (*Apis mellifera*). Apidologie.

[47] Vilmos P, Kurucz E (1998). Insect immunity: evolutionary roots of the mammalian innate immune system. Immunol Lett.

[48] Kavanagh K, Reeves EP (2004). Exploiting the potential of insects for in vivo pathogenicity testing of microbial pathogens. FEMS Microbiol Rev.

[49] Soares MPM, Elias-Neto M, Simões ZLP (2007). A cuticle protein gene in the honeybee: expression during development and in relation to the ecdysteroid titer. Insect Biochem Mol Biol.

